# A Multicenter, Prospective, Randomized Controlled Trial to Evaluate the Additional Benefit of a Multistrain Synbiotic (Prodefen®) in the Clinical Management of Acute Viral Diarrhea in Children

**DOI:** 10.1177/2333794X16679587

**Published:** 2016-11-18

**Authors:** Emilia García-Menor, Fátima García-Marín, Raquel Vecino-López, Gloria Horcajo-Martínez, María-José de Ibarrondo Guerrica-Echevarría, Pedro Gómez-González, Syra Velasco-Ortega, Javier Suárez-Almarza, Concepción Nieto-Magro

**Affiliations:** 1 Servicio de Pediatría del Hospital del Sureste de Arganda del Rey, Madrid, Spain; 2 Consulta de Pediatría del Hospital Virgen del Mar, Madrid, Spain; 3 Servicio de Pediatría del Hospital Universitario de Torrejón, Madrid, Spain; 4 Consulta de Pediatría de la Clínica Santa Elena, Madrid, Spain; 5 Consulta de Pediatría del Centro Médico del Val, Madrid, Spain; 6 Servicio de Pediatría del Hospital Universitario Juan XXIII, Tarragona, Spain; 7 ITF Research Pharma S.L.U., Madrid, Spain

**Keywords:** acute diarrhea, probiotic, synbiotic, Prodefen, children, tolerability, efficacy

## Abstract

This randomized, open-label study evaluated the additional benefits of the synbiotic Prodefen® in the clinical management of acute diarrhea of suspected viral origin in children between 6 months and 12 years of age. Study outcomes included the duration of diarrhea, the recovery from diarrhea, and the tolerability and acceptance of the treatment. The proportion of patients without diarrhea over the study period was greater in the synbiotic group than in the control group at all study time points, showing a statistically significant difference on the fifth day (95% vs 79%, p < 0.001). The duration of diarrhea (median and interquartile range) was reduced by 1 day in the synbiotic-treated patients (3 [2-5] vs 4 [3-5], p = 0.377). The tolerability of the treatment regimen, as evaluated by the parents, was significantly better in those receiving the synbiotic than in the control group. Overall, 96% of the parents of children receiving the synbiotic reported being satisfied to very satisfied with the treatment regimen. The results of this study indicate that the addition of the synbiotic Prodefen® is a well-tolerated and well-accepted approach that provides an additional benefit to the standard supportive therapy in the management of acute viral diarrhea in children.

## Introduction

Acute gastroenteritis (AGE) is a common illness that occurs worldwide. It has been defined by the European Society for Pediatric Gastroenterology, Hepatology, and Nutrition (ESPGHAN) as a decrease in the consistency of stools (loose or liquid) and/or an increase in the frequency of evacuations (typically ≥ 3 in 24 hours), with or without fever or vomiting. The most common cause of acute diarrhea is a viral infection, most frequently caused by rotavirus. It usually lasts less than 1 week and not longer than 2 weeks and occurs throughout the year, with a fall and winter predominance. Acute viral diarrhea accounts for most bouts of diarrhea in developed countries, with an incidence of 0.5 to 2 episodes per child per year in healthy European children younger than 3 years of age^1^ and is associated with substantial health care costs.^2^ The aim of treatment is to prevent or reverse dehydration, shorten the duration of the illness (important for preventing progression to persistent diarrhea, which is associated with adverse outcomes such as malnutrition), and reduce the period during which a person is infectious. The standard recommended treatment is based on diet and rehydration therapy.^2^

Probiotics have been defined as microorganisms that exert beneficial effects on human health when they colonize the bowel. They have been demonstrated to be efficacious for the treatment of diarrhea of suspected viral origin^3-5^ as well as for antibiotic-associated diarrhea^6,7^ by reducing the duration of the diarrhea and, additionally, as demonstrated in recent systematic reviews, reducing stool frequency.^3-5^ Although less well-demonstrated, some randomized clinical trials suggest that probiotics could also be effective for the prevention of nosocomial infections.^8,9^

Over the past few years, an abundance of probiotic-based products has appeared. Because probiotics are live microorganisms, the mere characterization of the strains contained in the product, independent of the evidence available of such strains, is insufficient to claim a beneficial effect. As such, it is well accepted that the efficacy of the specific strains, doses, and formulations in a certain product should be demonstrated. Consistent with this information, some clinical guidelines support the use of certain probiotic strains for the treatment of acute diarrhea in children.^1,2^

Synbiotics (a combination of probiotics and prebiotics) may be used for the management of acute viral diarrhea. However, it was concluded in the 2014 Guidelines for the Management of Acute Gastroenteritis in Children in Europe^1^ that none of the synbiotics studied at that time could be recommended until confirmatory data were available.

Prodefen® is composed of 1 × 10^9^ colony forming units (CFU) of the probiotic strains *Lactobacillus casei PXN 37, Lactobacillus rhamnosus PXN 54, Streptococcus thermophilus PXN 66, Bifidobacterium breve PXN 25*, *Lactobacillus* *acidophilus PXN 35*, Bifidobacterium *infantis PXN 27*, and Bifidobacterium *bulgaricus PXN 39*, in combination with the prebiotic fructooligosaccharide. It has been suggested that multistrain and/or multispecies probiotics may be more effective than monostrain probiotics because multistrain probiotics would have the hypothetical advantage of broad-spectrum coverage of possible enteropathogens and, furthermore, the advantage of multiple mechanisms of action. It has been suggested that the synergistic effects of different strains with specific properties may account for more than the sum of the separate health-promoting properties.^10,11^

Prodefen® has already been evaluated in a randomized clinical trial in a group of young children and has been shown to be beneficial in producing a significantly shorter duration of diarrhea and a reduction of the number of watery stools compared with placebo.^12^ However, as this study was performed in a very specific context (Iranian hospitalized children younger than 2 years of age), a new study was proposed to evaluate the benefits of the synbiotic Prodefen® in our ambulant pediatric population. The aim of this multicenter, prospective randomized and controlled study was to evaluate the additional benefits of the synbiotic Prodefen® when added to the standard treatment based on oral rehydration and/or diet in the clinical management of acute viral diarrhea, in children between 6 months and 12 years of age attending ambulatory outpatient clinics as well as emergency departments.

## Patients and Methods

### Participants

Participants were recruited from March 2014 to May 2015 in 3 outpatient clinics and 3 emergency departments in Spain. Consecutive patients aged 6 months to 12 years presenting with diarrhea, per the World Health Organization definition, that is, defined by the presence of 3 or more abnormally loose or watery stools in the previous 24 hours, were assessed for eligibility. The consistency of the stools was objectivized by the use of the Bristol Scale, with grades 5 to 7 defined as abnormally loose or watery stools.^13^ Patients were eligible if they visited the outpatient clinic or the emergency department for a diarrhea episode lasting less than 48 hours, if the diarrhea was suspected to have a viral origin, and if treatment with standard supportive therapy of oral rehydration and/or diet was to be initiated. Children were excluded if they had severe dehydration or malnutrition, presented visible blood in the stools, or had food allergies, lactose intolerance, milk protein allergy, immunodeficiency, or other underlying chronic (eg, inflammatory bowel disease) or acute (eg, pneumonia) diseases. They were also excluded if they were already receiving pharmacological treatment for acute diarrhea, had received antibiotic treatment and/or probiotics within the previous 7 days, or were participating in another study.

Written informed consent was obtained from the parents or legal guardians of the children. The study was approved by the ethics committee of the Hospital Universitario Juan XXIII (Tarragona, Spain) and Hospital Universitario de Getafe (Madrid, Spain).

### Interventions

After written informed consent was obtained, the patients were randomized to receive supportive treatment based on diet and/or oral rehydration therapy (control group) or supportive treatment supplemented with the synbiotic Prodefen® .

The children were assigned a consecutive number at each study site, according to the order of arrival, and children with an odd number were allocated to the synbiotic group and those with an even number to the control group. Children in the synbiotic group started treatment with Prodefen® at the pediatrician’s office or at the emergency room and were indicated to continue treatment at home using 1 sachet per day for 6 additional days.

This was an open-label study, and therefore, all people involved in the study, with the exception of the statistician in charge of performing the statistical analyses, knew the treatment assigned to every child.

### Study Evaluations

Every child was evaluated at baseline by the study investigator when they visited the outpatient clinic or the emergency room. During the visit, information about the demographic data, height and weight, physical examination, and relevant medical history was recorded. In addition, the investigator recorded the date and time of the first loose or watery stool, the frequency of stools in the previous 24 hours, the consistency of the stools according to the Bristol scale,^13^ the presence of other symptoms and signs, and the use of concomitant medications.

If the children met the selection criteria, they were randomized to the study arms, and their parents were instructed about the medication and use of the diary daily to record the number and consistency of stools according to the Bristol scale, as well as the frequency of other symptoms such as fever, vomiting, abdominal pain, and the presence of visible mucus in the stools; treatment adherence assessment; concomitant therapies; and adverse events. In addition, they were asked to note the time of the first normal stool. The parents were contacted by phone during the treatment phase on days 2, 4, and 7, so that the investigator could be informed of the course of diarrhea, treatments received, and the adverse events recorded in the diary for the corresponding period. In addition, on day 7, the parents were asked about the tolerability using an ad hoc 4-point Likert scale (from *very well tolerated* to *very poorly tolerated*), the perception of effectiveness using a 4-point Likert scale (from *very effective* to *poorly effective*), and in the synbiotic group, the satisfaction with the treatment using a 5-point Likert scale (from *very satisfied* to *very unsatisfied*). The parents were finally contacted on day 14, 7 days after the administration of the last dose (a window of 2 days was allowed if the scheduled phone call was foreseen during the weekend), so that any adverse event occurring in that period could also be recorded.

### Study Outcomes and Statistical Analyses

The primary objective was to evaluate the additional benefits of Prodefen® compared with the standard supportive therapy in the duration of acute viral diarrhea. Study outcomes were the duration of diarrhea (defined as the time in days from the first day of treatment to the last abnormal stool—loose or watery according to the Bristol scale), the recovery from diarrhea (defined as 2 consecutive days without diarrhea), the improvement of stool consistency, the cessation of other associated symptoms, and the tolerability and acceptance of the treatment.

All efficacy analyses were performed in those children who met the selection criteria and were followed up to the end of study. Safety analyses were performed in children who were randomized. Depending on the data distribution, baseline variables were described using the mean and standard deviation (SD) or the median and interquartile range (IQR) for quantitative outcomes and the absolute and relative frequencies for categorical outcomes. Statistical tests used for comparison included a chi-squared (χ^2^) test for categorical outcomes and Student’s *t* test for continuous outcomes, or nonparametric tests. All analyses were 2-tailed and were considered significant if *P* <0.05. All statistical analyses were performed with SPSS 21.0 (IBM Corp, released 2012; IBM SPSS Statistics for Windows, version 21.0, Armonk, NY: IBM Corp.).

The sample size was estimated to detect a difference of 1.20 (SD = 1.98) days in the duration of diarrhea, based on the results of a previous study carried out in Iran.^12^ For detecting this difference, with a power of 80%, a significance level of 5%, and an estimation of 15% of children lost to follow-up, 50 children were to be enrolled in each study group.

## Results

### Patient Disposition and Baseline Characteristics

A total of 101 patients were recruited, and 85 of them were evaluable, with 43 in the synbiotic group and 42 in the control group. The reasons for exclusion were the following: lost to follow-up (n = 10), withdrew synbiotic administration on the first day of treatment (n = 1), started antibiotic treatment (n = 1), started treatment with another probiotic (n = 1), showed blood in the stools (n = 1), was diagnosed with irritable bowel syndrome (n = 1), and had an intestinal invagination (n = 1).

The mean age of the children was 3.5 years (almost 50% were aged 6 months to 2 years), with a slight predominance of girls (58%; Table 1). The median number of stools was 5 in both study groups. The demographic and clinical characteristics (Table 1) were similar between the 2 study groups, with the exception of symptoms. A greater proportion of children in the control group were asymptomatic (19% vs 9%) and had fever (43% vs 28%) compared with the synbiotic group; in contrast, the frequency of abdominal pain was lower in the control group compared to the synbiotic group (55% vs 74%).

**Table 1.** Demographic and Clinical Characteristics.

	Total, n = 85	Control, n = 42	Prodefen®, n = 43	*P*
Age				
Mean ± SD, years	3.5 ± 3.2	3.6 ± 3.4	3.4 ± 3.1	0.803
6 months to 2 years, n (%)	41 (48.2)	19 (45.2)	22 (51.2)	0.584
3-5 years, n (%)	22 (25.9)	13 (31.0)	9 (20.9)	0.292
6-12 years, n (%)	22 (25.9)	10 (23.8)	12 (27.9)	0.666
Sex (boys), n (%)	36 (42.4)	17 (40.5)	19 (44.2)	0.729
Weight (kg), median (IQR)	14.0 (10.1-20.5)	14.4 (9.5-21.2)	14.0 (10.3-20.0)	0.937
Height (cm), median (IQR)	98.0 (81.3-117.3)	98.0 (82.0-116.5)	93.0 (81.0-118.0)	0.943
BMI (kg/m^2^), median (IQR)	15.6 (14.8-16.6)	15.7 (14.6-16.6)	15.4 (14.8-16.8)	0.939
Abnormal physical examination, n (%)	4 (4.7)	2 (4.8)	2 (4.7)	0.999
Number of stools				
Median (IQR)	5 (3-6)	5 (3-6)	5 (4-6)	0.824
≥5 stools, n (%)	47 (55.3)	23 (54.8)	24 (55.8)	0.992
Symptoms,^a^ n (%)				
No symptoms	12 (14.1)	8 (19.0)	4 (9.3)	0.175
Fever	30 (35.3)	18 (42.9)	12 (27.9)	0.149
Vomiting	25 (29.4)	13 (31.0)	12 (27.9)	0.758
Mucus on stools	24 (28.2)	11 (26.2)	13 (30.2)	0.679
Abdominal pain	55 (64.7)	23 (54.8)	32 (74.4)	0.058
Number of symptoms, median (IQR)	2 (1-2)	2 (1-3)	2 (1-2)	0.956

Abbreviations: IQR, interquartile range; BMI, body mass index.

a Reported by at least 5% of the children.

### Efficacy and Tolerability Outcomes

**Figure 1.**
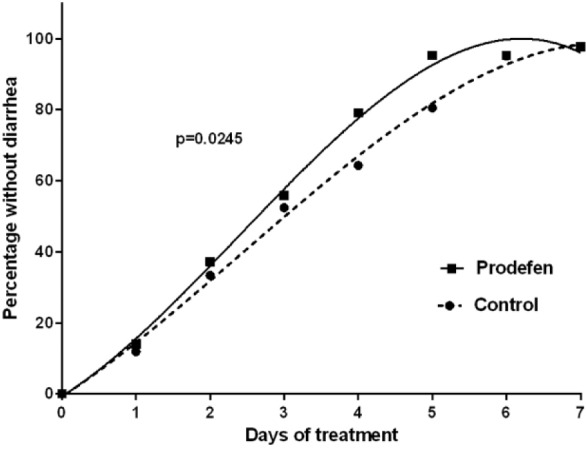
Children without diarrhea throughout the study. The data for both controland Prodefen®fitted to a third-order polynomial. *r*^2^ >0.996 for both curves. The resulting curves were compared using an extra sum-of-square *F* test (GraphPad Prism v.6.07).

The children receiving the synbiotic showed a more favorable evolution of the diarrhea than those in the control group (*P* = 0.02; Figure 1). The proportion of patients without diarrhea over the study period was greater in the synbiotic group than in the control group at all study time points, showing a trend toward statistical significance on the fourth day of treatment (79% vs 64%, *P* = 0.077) and a statistically significant difference on the fifth day (95% vs 79%, *P* < 0.001). Approximately 80% of the children had recovered from diarrhea after 4 and 5 days of treatment in the synbiotic and control groups, respectively. Similarly, approximately 95% of the children had recovered after 5 and 6-7 days of treatment in the synbiotic and control groups, respectively (Table 2).

**Table 2.** Children Without Diarrhea Throughout the Study,n(%).

	Control (n = 42)	Prodefen® (n = 43)	*P* Value
Day 1	5 (11.9)	6 (14.0)	0.715
Day 2	14 (33.3)	16 (37.2)	0.593
Day 3	22 (52.4)	24 (55.8)	0.580
Day 4	27 (64.3)	34 (79.1)	0.077
Day 5	33 (78.6)	41 (95.3)	<0.001
Day 6	39 (92.9)	41 (95.3)	0.360
Day 7	40 (95.2)	42 (97.7)	0.616

**Figure 2.**
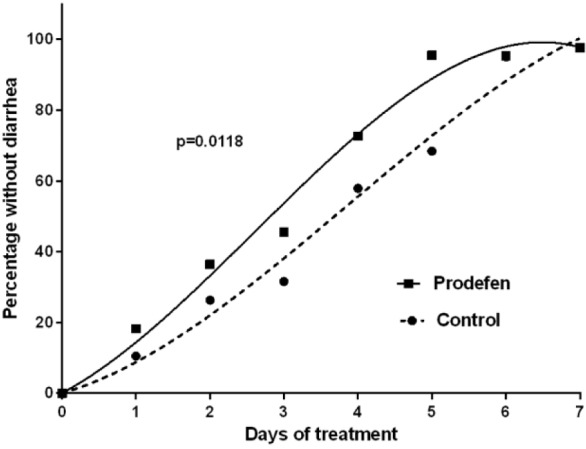
Children between 6 months and 2 years old without diarrhea throughout the study. Data for both the control and Prodefen®fitted to a third-order polynomial. *r*^2^ >0.985. The resulting curves are compared using an extra sum-of-square *F* test (GraphPad Prism v.6.07).

This difference in the recovery from diarrhea between the synbiotic and control groups was likewise evidenced in the evaluation of the duration of diarrhea (median and IQR), which was reduced by 1 day in the synbiotic-treated patients (3 [2-5] vs 4 [3-5], *P* = 0.377). When analyzed by the children’s age, the synbiotic group had a significantly shorter duration of diarrhea than the control group in children aged 6 months to 2 years, with the duration shortened by 2 days (3 [2-4] vs 5 [3-5], *P* = 0.034). Additionally, in these children, the proportion of those without diarrhea was greater at all study time points in the synbiotic group than in the control group, with almost all of the children in the synbiotic group and two-thirds of those in the control group having recovered at day 5 (95.5% vs 68.4%, *P* = 0.036; Table 3). The evolution of the diarrhea in these children aged 6 months to 2 years was also more favorable in those receiving the synbiotic than in those in the control group (*P* = 0.01; Figure 2).

**Table 3.** Children Between 6 Months and 2 Years Old Without Diarrhea Throughout the Study, n (%).

	Control (n = 19)	Prodefen® (n = 22)	*P* Value
Day 1	2 (10.5)	4 (18.2)	0.668
Day 2	5 (26.3)	8 (36.4)	0.524
Day 3	6 (31.6)	10 (45.5)	0.522
Day 4	11 (57.9)	16 (72.7)	0.346
Day 5	13 (68.4)	21 (95.5)	0.036
Day 6	17 (89.5)	20 (90.9)	0.999
Day 7	18 (94.7)	21 (95.5)	0.999

The number of stools (median [IQR]) was slightly lower in the children receiving the synbiotic than in the control group on days 5 (1 [1-2] vs 2 [1-3], *P* = 0.105) and 6 (1 [1-2] vs 1.5 [1-2], *P* = 0.511) of treatment. As for the stool consistency, no significant differences were found between treatments. However, the difference between the number of watery stools on the fifth day of treatment compared to baseline was significantly different (−5 vs −3, *P* = 0.031) in the synbiotic group compared to the control group.

The number of children in the synbiotic group who required an additional visit to the pediatrician after randomization was almost half that of the control group (6 [14.0%] vs 11 [26.2%], *P* = 0.158). The frequency of adverse events or the presence of other symptoms was similarly low in both study groups: fever (11.5% vs 4.1%), vomiting (1.9% vs 4.1%), mucus in the stools (1.9% vs 2.0%), and abdominal pain (1.9% vs 2.0%), for synbiotic-treated and control patients, respectively.

### Parent-Reported Outcomes

**Figure 3.**
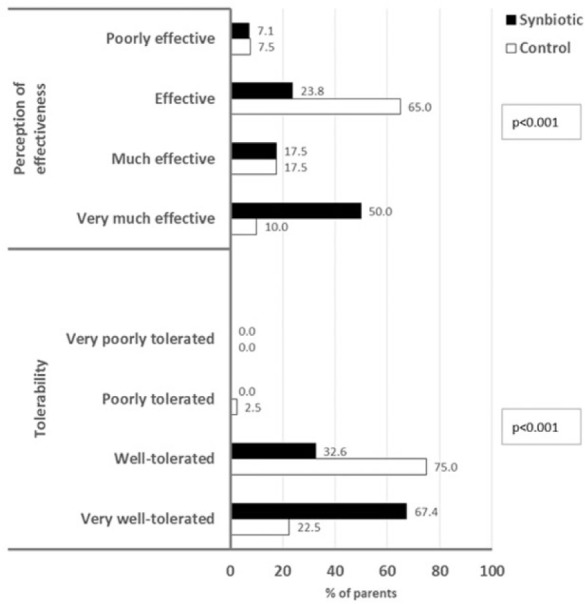
Tolerability and effectiveness as reported by the children’s parents.

**Figure 4.**
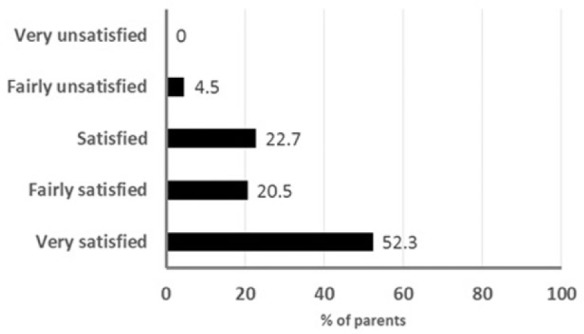
Treatment satisfaction as reported by the children’s parents.

The tolerability of the treatment regimen, as evaluated by the parents, was significantly better in those receiving the synbiotic than for those in the control group (Figure 3); 67% of the parents of the children receiving the synbiotic considered that the treatment regimen was very well tolerated compared with 23% of parents of children receiving the standard treatment alone (*P* < 0.001). Similarly, the perceived efficacy was significantly greater among the parents of children receiving the synbiotic than the parents of the control group (Figure 3), with 68% of the parents considering the synbiotic very or quite effective compared with 28% of the parents of children receiving the standard treatment alone. Overall, 96% of the parents of children receiving the synbiotic reported being satisfied to very satisfied with the treatment regimen (Figure 4).

Compliance with diet (67% vs 67%, *P* = 0.937) and with oral rehydration therapy (52% vs 40%, *P* = 0.231) was not different among the synbiotic-treated children compared with the control group. Compliance with the synbiotic was high (90%), and 41 parents (95.3%) were willing to use the product again.

## Discussion

This randomized trial shows that the addition of Prodefen® to the standard treatment with diet and oral rehydration shortens the duration of diarrhea and is very well tolerated in children with acute diarrhea of suspected viral origin. These findings are in line with current scientific evidence on the benefits and safety of probiotics in the treatment of acute infectious diarrhea^3^ and provide new evidence on the benefits of a synbiotic in the management of children with this pathology.

In this trial, the efficacy of Prodefen® was studied by analyzing the duration of the diarrhea after the beginning of treatment, evaluated by the number of days with diarrhea and the percentage of patients without diarrhea at various study time points. The children who received the synbiotic in addition to the standard therapy with diet and/or oral rehydration showed a more favorable clinical evolution than those who received the standard therapy alone. The addition of the synbiotic accelerated recovery from diarrhea by approximately 1 day, as evidenced in the percentages of children receiving synbiotics without diarrhea after 4 and 5 days, which matched those in the control group after 5 and 6 days of treatment. Notably, practically all children receiving Prodefen® recovered in 5 days, whereas this happened 2 days later in the children receiving the standard treatment.

An important question arises as to whether these differences in the evolution of the diarrhea are clinically relevant. In this study, by day 4, the difference in the proportion of patients who did not have diarrhea was 15%, which is equivalent to a number needed to treat (NNT) of 6.6, and by day 5, the difference between the 2 regimens was 17%, equivalent to a NNT of 5.9. These figures represent a clinically relevant effect. Although these study outcomes are subjective and, therefore, subject to an evaluation bias, the excellent results attributed to the synbiotic by the parents regarding the perceived efficacy and satisfaction support the clinical relevance of the objective diarrhea-related outcomes.

From a clinical standpoint, this reduction in the time to resolution is of relevance not only because of the more direct impact of the diarrhea episode in the children’s health and well-being, but also because of the impact that prolonged episodes might have on intestinal dysbiosis, which could lead to eventual intolerances and malabsorption that, although transient, would have a negative nutritional influence on the patients. Treatment with the synbiotic, apart from accelerating children’s recovery, might palliate potential adverse consequences of this pathology.

Significantly, Prodefen® reduced the duration of the diarrhea by 2 days in children younger than 2 years. These results are particularly relevant in infants because this is an especially vulnerable population, often debilitated when affected by this condition and more susceptible to dehydration and related symptoms than are older children. In this scenario, the social impact of this 2-day difference for parents and caregivers (earlier school attendance, less day care and parental absenteeism, etc) should also be particularly valued.

The results obtained in this study are consistent with those reported with this preparation in a different setting. Allahverdi et al,^12^ in a randomized double-blind, placebo-controlled trial undertaken in Iranian children between 1 and 5 years old with acute viral diarrhea, found that the addition of this synbiotic to dietary and hygienic measures was associated with a reduction of 2 days in the duration of diarrhea compared with placebo. Yala et al.^14^ evaluated the addition of a similar synbiotic to a standard treatment with oral rehydration and zinc supplementation in Philippine children aged between 2 months and 2 years with AGE who were admitted to the hospital. These authors did not report the impact of the synbiotic on the duration of diarrhea, but it is inferred that it was shortened because the synbiotic significantly reduced the frequency of stools compared with the standard treatment alone as early as the second day and reduced the hospital stay by 1 day.^14^ The results of our study are also consistent with those reported with probiotics in recent meta-analyses.^3,15^ In a study carried out within the Cochrane Collaboration that included 56 trials undertaken in young children with acute infectious diarrhea who received a wide range of probiotics and dosages, the duration of diarrhea was shortened by 1 day compared with placebo or no use of probiotic.^3^ Similar results were reported in another meta-analysis of studies undertaken with *Saccharomyces boulardii* in patients with acute infectious diarrhea of various etiological causes, where the patients receiving the probiotic had a difference in the duration of diarrhea of 1 day compared with the placebo group.^15^

We did not find a significant difference between the 2 study groups in the frequency of stools or in their consistency. Some meta-analyses have found that the use of probiotics for acute diarrhea is also associated with a reduction in the frequency of stools,^3,5,15^ but others failed to find a difference in this outcome.^4^ On the other hand, the number of stools in our study was relatively low (a median of 5 [IQR = 3-6] at baseline); therefore, the limited effect of the synbiotic on the number of stools in our study could be a result of a floor effect.

No safety concerns arose during the study. The synbiotic was very well tolerated, as shown by the low frequency of adverse events or related symptoms, and its efficacy was highly rated in the overall evaluation of the parents. Notably, the children who required additional visits to their pediatrician were almost halved (in percentage) in the synbiotic group compared with the control group, which indirectly indicates the benefits of the synbiotic in the management of the pathology, because usually additional visits occur when children do not recover in a reasonable period of time, usually perceived by the parents to be 4 to 5 days.

Our study has a number of limitations, with the major limitation being the lack of blinding for the investigators. As stated above, although the evaluation of the diarrhea is an objective measure and is not in principle affected by the investigator’s bias, this lack of blinding could have overestimated the effect of the synbiotic regarding the more subjective outcomes, such as the parents’ perceived efficacy, tolerability, and satisfaction. In addition, including a placebo was advised against by the investigators, who considered that most parents would not accept the administration of a placebo to their children. In contrast, the major strength of our study is that it was carried out with a specific product under similar conditions to usual clinical practice, increasing the applicability of our results.

Overall, the results of this study indicate that the addition of the synbiotic Prodefen® is a well-tolerated and well-accepted approach that provides an additional benefit to standard supportive therapy in the management of acute viral diarrhea in children. Because diarrhea accounts for a significant quantity of pediatric outpatient visits and hospitalizations as a result of poor disease resolution, besides obvious health benefits to the child, a reduction in the time to resolution may have important public health implications in terms of missed work and lost revenue, and school and day care absenteeism.

## Author Contributions

EG-M: Contributed to the conception and design of the study, acquisition and analysis of data, and writing of the first draft.

FG-M: Contributed to the acquisition, analysis, and interpretation of data

RV-L: Contributed to the acquisition of data.

GH-M: Contributed to the acquisition of data.

M-JIG-E: Contributed to the acquisition, analysis, and interpretation of data.

PG-G: Contributed to the acquisition of data.

SV-O: contributed to the analysis of data and critical review of the manuscript.

JS-A: Contributed to the conception and design of the study, analysis and interpretation of data, and critical review of the manuscript.

CN-M: Contributed to the conception and design of the study, analysis and interpretation of data, and critical review of the manuscript.
